# I Am the 1 in 10—What Should I Eat? A Research Review of Nutrition in Endometriosis

**DOI:** 10.3390/nu14245283

**Published:** 2022-12-11

**Authors:** Małgorzata Piecuch, Jagoda Garbicz, Martyna Waliczek, Jolanta Malinowska-Borowska, Piotr Rozentryt

**Affiliations:** Department of Toxicology and Health Protection, Faculty of Health Sciences in Bytom, Medical University of Silesia in Katowice, 41-902 Bytom, Poland

**Keywords:** endometriosis, nutrition, anti-inflammatory diet, antioxidants, pelvic pain

## Abstract

Endometriosis is a chronic, painful, estrogen-related inflammatory disease that affects approximately 10% of the female population. Endometriosis has a significant negative impact on quality of life. Nutrition may be involved in the development and severity of endometriosis. The purpose of this paper is to discuss in detail the nutritional recommendations for patients with endometriosis. This article discusses the importance of nutrients such as polyphenols, vitamins C, D and E, PUFAs, and iron in the development of endometriosis. Alternative diets, such as the Mediterranean, anti-inflammatory, vegetarian, low-nickel and low-FODMAP diets, have also been presented in the context of their potential beneficial effects on the course of endometriosis.

## 1. Introduction

Endometriosis (EMS) is a chronic, estrogen-related inflammatory condition, which affects approximately 10% of the female population [1,2,3]. In this ailment, endometrium-like tissue develops outside the uterine cavity, most often in the pelvic areas (fallopian tubes, ovaries and peritoneum as well as bladder, ureters, and intestines), and less often in the lungs, brain, and retina [2,4]. Endometriosis is clinically characterized by chronic pain in the abdomen, dysmenorrhea, dysuria, dyschezia and dyspareunia. Infertility is more common in women with EMS (10–15%) than in the general population (9%) [2,4]. A variety of symptoms may exert a considerably deleterious effect on women’s standard of living [4,5]. A large problem is the waiting time for diagnosis—in Europe, women wait an average of 7–12 years for a correct diagnosis of endometriosis. Difficulties in the recognition and therapy of endometriosis also result from the lack of clear recommendations. Diagnostic laparoscopy with biopsy, previously considered the gold standard in identification of EMS, has been presented in the latest ESHRE guidelines (2022) as among the possible options, not necessarily the best and required for an accurate diagnosis [6]. Endometriosis is treated surgically and pharmacologically. The care of a dietitian and urogynecological physiotherapist are both essential for the health condition and improvement of the comfort of life of women with EMS.

The pathogenesis and etiology of endometriosis remain poorly understood. The list of pathogenic factors contributing to the progress of endometriosis include retrograde menstruation, genetic profile, hormonal activity, inflammation, immune dysfunction, oxidative stress, organochloride burden, metaplastic processes and apoptosis suppression [7]. It has been attested that disproportions between the invasive, adhesive and proliferative qualities of the endometrium’s cells and the increase in a production of pro-inflammatory cells are responsible for the development of the disease [8]. 

Among the factors contributing to the appearance of ectopic endometrial implants in the peritoneal cavity is an immune dysfunction which allows abnormal tissues, otherwise naturally eliminated, to survive. The appearance of implants results in increased oxidative stress and the gradual development of inflammation. Macrophages in the peritoneum produce growth and angiogenesis factors and inflammatory cytokines which may be responsible for maintaining the disorder and impairing reproductive function [9].

The latest studies demonstrated that EMS is an angiogenesis-dependent disease [10]. Angiogenesis has a significant impact on the progress of endometrioid tissue. Highly responsive angiogenic factor is a vascular endothelial growth factor (VEGF), which promotes the angiogenesis in physiological and pathological conditions [10,11]. 

There are four EMS pathological stages—I—minimal, II—mild, III—moderate, and IV—severe—taking into account, among others, the depth and location of implants and the presence and location of adhesions [12].

Endometriosis shows some similarities with malignant tumors—endometriosis tissues nest in ectopic ones, grow invasively and damage the structures of adjacent tissues. Patients with endometriosis face the increased risk of contracting some type of cancer, including ovarian cancer [13,14,15], non-Hodgkin’s lymphoma, endocrine cancer, brain tumors and endometrial and breast cancer [15,16]. 

Differences in the incidence of endometriosis in different parts of the world may suggest that environmental factors, lifestyle, and diet may be etiological factors in addition to genetic determinants [16].

Diet, as a modifiable risk factor, may be involved in the progression and severity of EMS. The effect of nutrition on endometriosis may result from changes in estrogen and prostaglandin metabolism, smooth muscle contractility or inflammation [17].

In endometriosis, as an estrogen-related disease, estrogens are key factors of endometrial cellular growth [18]. Estrogen has the ability to promote, survive, migrate, adhere and proliferate endometrial stroma and epithelial cells [16]. Certain nutrients can inflect endogenous hormone metabolism as well as imitate or demonstrate estrogen. The consumption of phytoestrogen, fatty acids, coffee, alcohol and fiber has been connected to endogenous estrogen levels [19]. 

## 2. BMI and Endometriosis

Obesity has a detrimental effect on the endometrium and is an important component for the progression of endometrial cancer. However, epidemiological data show an inverse relationship between BMI and the incidence of endometriosis. The causes of this obesity paradox stay unexplained and more research is necessary [20]. An inferior BMI is not only a EMS development risk, but also a predisposing factor for severe endometriosis [21]. Chronic pelvic pain (CPP) is noted for its association with a lower BMI [20,21,22]. A meta-analysis of 11 studies found that a higher BMI coincided with a lower EMS risk. A 33% decrease in endometriosis risk was observed in correlation with every 5 kg/m^2^ increase in BMI [23,24]. It should be taken into account that BMI is not the most reliable indicator of obesity. The obesity paradox hypothesis should be further explored based on the body composition analysis and not exclusively on the basis of BMI. Some explanations for the reverse epidemiology in endometriosis include decreased appetite and lower calorie and nutrient intake due to gastrointestinal complaints, chronic endometriosis-related stress and pain, and ovulatory suppression/irregular menstruations in obese women [24]. 

## 3. Vegetables and Fruit

### 3.1. Polyphenols

Numerous studies provide solid evidence of positive health effects of eating a diet rich in vegetables and fruits, because of the existence of the bioactive plants’ composites, especially polyphenols. Polyphenols have proven anti-cancer, anti-inflammatory, anti-atherosclerotic, antioxidative and anti-hypertensive properties [25]. The anti-inflammatory potential of natural dietary polyphenols might be used in the management of endometriosis as an inexpensive, non-toxic and readily available agent [26]. Several studies indicate an inverse correlation between polyphenol application, such as phytoestrogens and female cancer risk [27,28,29]. 

### 3.2. Phytoestrogens

Phytoestrogens (PE) are naturally occurring plant compounds which are structural and functional homologies with estrogen. There are three classes of PE: lignans, stilbenes, and flavonoids [18]. Phytoestrogens are found in vegetables, fruits, tea, grains, beans, sprouts, soybean, oilseeds and cabbage. PE are very similar in structure to estrogen, and, owing to that similarity, they can function as weak estrogenic factors interfering with molecular and hormonal signaling, which prevents the advancement of non-insulin-dependent diabetes, cancer, obesity, cardiovascular diseases and negative menopausal symptoms [18]. Phytoestrogens could link to estrogen receptor (ER) and affect ER-mediated responses [16]. A lot of case–control studies investigated connection in the midst of PE ingestion and endometriosis risk. A comparison of PE consumption by 78 patients with laparoscopically proven EMS versus 78 healthy women by Youseflu et al. shows that the intake levels of isoflavones and lignans were inversely correlated with endometriosis risk [19]. In a systematic review by Bartiromo et al., 19 out of 22 studies indicated the capability of phytoestrogens to generate proapoptotic, anti-inflammatory, anti-proliferative outcomes on cultured cells [18].

### 3.3. Resveratrol

Among the most known and tested polyphenols, whose beneficial influence on health was proven in in vitro and in vivo models and in clinical tests, is resveratrol (RSV). More than 70 types of plants and plant-based products, principally black and red grapes, red wine, berries and nuts, comprise RSV, which is a phytoalexin and a phytoestrogen [26,30,31]. 

A number of in vitro tests [32,33,34,35,36,37] have investigated the impact of RSV on endometrial and endometriotic cells and have demonstrated a pro-apoptotic function and inhibitory effect on cell proliferation and invasive growth. These effects can be partially explained by inhibitory effect on the expression of insulin-like growth factor-1 (IGF-1), surviving and hepatocyte growth factor (HGF), while its effects on estrogen are more complex.

In vivo models of EMS have confirmed both the pro-apoptotic, anti-invasive role and also an inhibitory impact on angiogenesis, which is related to a drop in the expression of pro-invasive, pro-angiogenic and pro-inflammatory factors, particularly interleukin-6 (IL-6) and interleukin-8 (IL-8) [34,38,39,40]. 

The clinical studies [41] on the combination of oral contraception with RSV have informed potentially positive results in a treatment of endometriosis-related symptoms: decreased dysmenorrhea and pain relief; however, the number of studies is insufficient [31]. 

A clinical trial of Mendas et al. showed no positive effects of resveratrol consumption [42]. RSV proved its efficiency either alone or in combination with other substances used in EMS therapy such as statins or leuprolide acetate [26]. 

### 3.4. Vitamins C

Vitamin C, among the most important antioxidatives, strongly reduces free radicals. Humans are incapable of synthesizing vitamin C, so it should be provided with food or in the form of supplements/medications. Peppers, citrus, kiwi, broccoli, tomatoes, potatoes and strawberries are some of the products renowned for their high content of vitamin C [43]. 

Hoorsan et al., in their experiment on mice, suggested that vitamin C has considerable importance for improving the fecundity function of ovaries and reducing the induction and growth of endometrial implants [44].

A randomized, triple-blind placebo-controlled clinical study of Amini et al. with the supplementation of vitamins C and E confirmed the effect of lowering the systemic indicators of oxidative stress in patients with EMS [45].

Ansariniya et al., in a lab trial, determined the influence of vitamins C and E on VEGF gene expression and production in peritoneal macrophages of women with endometriosis in comparison with the control group. It was found that vitamins C and E, at different incubation times and concentrations, changed the expression of the VEGF gene in the peritoneal macrophages but they had no effect on the production of VEGF [11].

## 4. Spices and Herbs

According to the definition proposed by the Food and Drug Administration (FDA), spices are: “aromatic vegetable substances, in the whole, broken, or ground form, whose significant function in food is seasoning rather than nutrition” [46]. Herbs and spices, such as oregano, rosemary, thyme and parsley, contain high levels of polyphenols, especially phenolic acids and flavonoids [47]. There are many herbs with known or potential significant anti-inflammatory activity. The spices with the most known anti-inflammatory effect are: thyme, oregano, basil, rosemary, mint, sage, curcuma, dill, cinnamon, parsley, clove, lemon grass, nutmeg, ginger, fenugreek, pepper and chili pepper [47,48,49,50,51,52,53]. 

Many of the anti-inflammatory substances (such us capsaicin, gingerol, and curcumin) in spices and herbs inhibit one or more of the steps linking pro-inflammatory stimuli with cyclooxygenase (COX) activation [47].

Curcuma (also known as turmeric) is a spice with proven anti-inflammatory and anti-cancer properties. It reduces the concentration of estrogens, TNF alpha and interleukin mediators, accelerates cellular apoptosis, and inhibits angiogenesis [49,54,55]. Consuming turmeric in conjunction with black pepper (containing piperine) increases the bioavailability of curcumin by up to 2000% [56]. 

In the studies of Fadin et al., a marked reduction in pain associated with endometriosis and the use of smaller amounts of non-steroidal anti-inflammatory drugs was observed in women supplemented with 200 mg of quercetin, 210 g of Curcuma longa’s dry extract and 150 mg of acetylcysteine for 2 months [57]. 

## 5. Tea and Coffee

White and green tea contain more catechins (substances classified as polyphenols) with strong antioxidant properties compared to black tea. White tea comprises minerals, proteins, amino acids, caffeine, gallic acid and catechins [58]. 

Meta-analysis suggests that caffeine intake of less than 300 mg/day is not linked with a higher EMS development risk. Higher caffeine consumption may be related to the development of the disease, but there is a shortage of well-designed, large-scale clinical trials to explain this relationship and the relevance of caffeine in the pathophysiology of EMS [59].

Caffeic acid, a polyphenol present, e.g., in coffee, some vegetables and legumes, reduces oxidative stress, which can alleviate complications from endometriosis [60].

## 6. Dairy Food

Dairy is a good source of progesterone, estrogen, calcium, vitamin D, anti-inflammatory and anti-tumorigenic ingredients, polyunsaturated fatty acids (PUFAs) and butyric acid [19].

There are some hypotheses regarding potential biochemical and physiological effects of dairy on the risk of developing EMS. Among them is that a high consumption of calcium-rich dairy products may reduce inflammatory and oxidative stress by decreasing inflammatory factors: tumor necrosis factor-a (TNF-a), reactive oxygen species and IL-6 [17]. An inverse relationship between the level of vitamin D and C-reactive protein was observed in atherosclerosis vascular disease and diabetes mellitus, which may suggest a similar relationship in endometriosis [17]. 

In a case–control study of Iranian Women, it was found that higher dairy intake is connected with a reduced risk of EMS [19]. Additionally, the longitudinal cohort study by Nodler et al. demonstrated that adolescents who consumed larger portions of dairy, including yoghurt and ice cream, had a lower rate of endometriosis recognition in adulthood [17]. 

A Dose–Response Meta-Analysis by Qi et al. showed that significant effects of reducing endometriosis were obtained with a daily consumption of ≥3 servings of dairy products. That study also analyzed a relationship between endometriosis and particular types of dairy—the heavy consumption of high-in-fat dairy and cheese might result in a decrease of EMS risk, while high butter consumption may lead an increased risk of endometriosis [61]. 

## 7. Fish

Fish oil has been shown to decrease circulating levels of series 2 prostaglandins and decrease dysmenorrhea and inflammatory symptoms [62]. 

### 7.1. Omega 3 and Omega 6

Polyunsaturated fatty acids (PUFAs) are fatty acids containing a minimum of two double bonds. Omega 3 (*n*-3) and omega 6 (*n*-6) PUFAs are found in fatty fish and seed/vegetable oils [63]. 

Akyol et al., in a randomized, single-blind, prospective and controlled experimental trial on female rats, observed that omega 3 caused a considerable recession of endometriotic implants [64]. In a clinical trial by Nodler et al., a reduction in pelvic pain in women with endometriosis who complemented fish oil rich in omega 3 was noted; however, a similar result was noticed in the placebo group [65]. 

A cross-sectional research by Hopeman et al. [63] examined samples and data from women undergoing in vitro fertilization (IVF) with surgically proven endometriosis and from women without endometriosis to ascertain if there is a relationship with serum omega 3 PUFAs α-linolenic acid (ALA), eicosapentaenoic acid (EPA) and docosahexaenoic acid (DHA) or *n*-6 PUFAs linoleic acid (LA) and arachidonic acid (AA) and endometriosis. This study showed that patients with higher serum EPA levels were at 82% less risk of EMS development in contrast with patients with low EPA levels, while no association between total PUFAs, total omega 3 PUFAs, or total omega 6 PUFAs and EMS was found [63]. 

In the controlled experimental study of female rats, Pereira et al. demonstrated that the omega 6/3 and omega 9/6 nutraceuticals diminished pain associated with endometriosis, but did not improve fertility [66]. 

### 7.2. Vitamin D

Fatty fish and cod liver oil contain vitamin D3 (cholecalciferol) and its metabolite, 25(OH)D3, a fat-soluble secosteroid hormone that plays a significant role as a immunomodulatory and anti-proliferative mediator. The best source of vitamin D is skin synthesis, which occurs under the influence of the activity of ultraviolet B radiation [43,67].

Vitamin D receptors and metabolizing enzymes are found in the endometrium and ovaries of women with and without EMS, and it is assumed that cholecalciferol might affect immune cells in the local environment [43].

In the randomized, double-blind, placebo-controlled study in 60 women with endometriosis, it was shown that regular cholecalciferol intake in women resulted in a considerable increase in total−/HDL-cholesterol ratio and hs-CRP and TAC levels and pelvic pain, but this had no influence on metabolic profiles and further clinical manifestations [68].

In an evidence-based critical appraisal of vitamin D in reproduction, Lagana et al. suggested that the supplementation of vitamin D should be carefully evaluated, considering the pleiotropic actions in diverse microenvironments of the body as well as the different sources of dietary intake and synthesis [67]. EMS is related to a normal or high 25(OH)D reserve, which may be due to an equalizing mechanism controlling inordinate local inflammation commonly noticed in women with endometriosis. However, endometriosis patients report a meaningful decrease in the pelvic pain during supplementation of vitamin D [69].

## 8. Meat

Nutrition may correspond to the risk of EMS by influencing steroidal hormones. Red meat has been shown to increase estradiol concentrations and reduce sex hormone-binding globulin (SHBG). Estrogen increases the synthesis of prostaglandins, and positive feedback for local estrogen and prostaglandins could aid the proliferative and inflammatory features of EMS [62].

The Nurses’ Health Study II (NHSII) prospective cohort has shown that the consumption of red meat, both processed and unprocessed, was correlated with a greater risk of endometriosis: women eating > 2 portions of red meat a day had a 56% greater risk of laparoscopically proved endometriosis in comparison to women eating ≤ 1 portion a week. This investigation among premenopausal US nurses insinuates that reduced red meat consumption could be a strategy for reducing the risk of endometriosis, especially for women with pain symptoms [62].

Excessive ingestion of red meat is related to greater levels of estradiol and estrone sulfate, resulting in the persistence of the disease [70,71].

Different results were obtained by Ashrafi et al., who showed a decreased endometriosis risk for those consuming 4–6 portions of meat per weak compared to those who consume 0–3 portions of meat per week [72].

### 8.1. Iron (Fe)

Iron is an indispensable component for cell survival and its insufficiency is a known risk factor for many reproductive dysfunctions. Iron-deficiency anemia is not often diagnosed in patients with EMS, despite heavy menstrual bleeding and chronic inflammation of the abdominal cavity. Atkins et al. [73] examined non-human primate models (macaques) with and without endometriosis and observed anemia in almost half of the macaques with endometriosis and also an abnormal hematogram—decreased RBC counts and serum hepcidin and increased MCV and percentage of reticulocytes. Reduced levels of hepatic and bone marrow Fe were found and increased intestinal expression of ferroportin 1, a mediator of iron absorption, which indicates that though there was greater iron content in the diet, intestinal iron absorption did not equalize for the Fe loss. Atkins et al. concluded that iron stores should be rated in women with EMS, even without apparent clinical hints of anemia [73].

Shu-Wing Ng et al. proposed that abnormal eutopic endometrium is marked by impedance to ferroptosis, which allows cell spread via retrograde menstruation to implant and establish endometriotic lesions within the abdominal cavity. Deregulated iron homeostasis can be crucial in the subsequent pathophysiology of endometrial lesions with local iron overburden and inflammation [74].

### 8.2. Iron Overload and Infertility

The peritoneal fluid (PF) of women with EMS is iron overloaded, with negative results on early embryo evolution by an unknown mechanism. Iron overload in endometriosis PF decreased GPX4 expression, disrupted blastocyst formation and induced lipid peroxidation, suggesting that iron overload causes embryotoxicity and induces ferroptosis [75]. The results of the experiment on mice by Chen et al. implicated that excess iron found in the peritoneal fluid of women with endometriosis probably participates in endometriosis-associated reproductive failure [76].

The results of an experiment by Li et al. possibly suggested that transferrin deficiency and iron overburden in follicular fluid from severe endometriosis leads to oocytes dysmaturity, which can contribute to infertility [77].

## 9. Alternative Diets

### 9.1. Antiinflammatory Diet

Endometriosis is a chronic inflammatory disease. A typical Western diet, high in fat and high in calories, promotes inflammation. On the other hand, many fruits, vegetables and mineral-rich foods, such as traditional Mediterranean and Japanese foods, have anti-inflammatory results. To assess the inflammatory potential of a diet, the Dietary Inflammation Index (DII) is used. The higher the DII score, the greater the diet’s proinflammatory potential. The higher the negative DII value, the more anti-inflammatory the diet is. Kyozuka et al. [78] indicate that an anti-inflammatory diet is appropriate for patients with endometriosis, not only because of the reduction in inflammation associated with endometriosis, but also because of the positive effect of an anti-inflammatory diet in the pre-pregnancy period on fertility and a reduction in pregnancy and perinatal complications.

### 9.2. The Mediterranean Diet

The Mediterranean diet (MD) ranks high among the healthiest diets worldwide. The MD is mostly a plant-based diet, dominated by vegetables, fruits, dry legume seeds, and nuts, with a moderate intake of dairy product and fish and low intake of red meat and wine [79]. The MD has many benefits in gynecological diseases [80] as well as in the prevention of the majority of non-communicable diseases, including cardiovascular diseases and cancers [81]. In a single-arm study by Ott et al., it has been shown that that eating fresh vegetables and fruits, whole meal products, pods, soy products, fish rich in fat, white meat and cold-pressed oils as well as decreasing the consumption of red meat, sugary drinks, animal fats and sweets improved the general well-being of women with endometriosis and reduced pain, dyspareunia, dysmenorrhea and dyschezia [82].

### 9.3. Vegetarian/Vegan Diet

Plant-based diets are characterized, on the one hand, by a large intake of vegetables, fruits and herbs, and, on the other hand, by the elimination of the consumption of meat and animal fats. This coincides with dietary recommendations for women with endometriosis, so it seems reasonable for patients to follow such diets.

Studies have indicated that vegetarian diets can lead to increased levels of sex hormone-binding globulin (SHBG) and decreased estrogen levels in women. This is followed by a decrease in estrogenic stimulation of the endometrium and a reduction in the proliferation of prostaglandin-producing tissues [72].

### 9.4. Low-Nickel Diet

Nickel in foods such as tomatoes, beans, whole grains, nuts, shellfish, garlic, onion, soy, corn and tea may cause allergic contact mucositis (ACM) in some people, which causes symptoms similar to irritable bowel syndrome (IBS). These symptoms and ACM are also seen in patients with endometriosis. An open-label pilot study [83] showed that a low-nickel diet might significantly decrease not only all gastro- and extraintestinal but also gynecological symptoms [83].

### 9.5. Low-FODMAP Diet

A low-FODMAP diet is low in fermentable oligosaccharides, disaccharides, monosaccharides and polyols (FODMAP), carbohydrates found in grains, fruits and vegetables. FODMAPs are small molecules, poorly absorbed and readily fermentable by bacteria. Through their gas production and an osmotic action, they can cause intestinal luminal distension, inducing bloating and pain in people with visceral hypersensitivity, which occurs in patients with IBS and also in women with endometriosis [84,85]. Moore et al. [85] found that the low-FODMAP diet seems to be beneficial for patients with endometriosis and gut symptoms. It should be emphasized that this diet, due to its nutritional limitations, is not suitable for long-term use. As a standard, it is used for a few weeks, then subsequent food products are gradually restored, eliminating only those that aggravate intestinal symptoms. There are few studies describing the long-lasting effects of using the low-FODMAP diet for a long time. It is suggested that the dietitian-supervised low-FODMAP diet consisting of three stages—restriction, reintroduction, and personalization—could be safely used over prolonged periods of time [86].

### 9.6. Gluten-Free Diet

Gluten-free diet may alleviate pain due to the obstruction of gluten-mediated immunomodulation and the inflammatory response by inflecting the cytokine system. In a retrospective study of 330 women by Marziali et al. [87], 75% of the patients reported a considerable mitigation of their pain symptoms. Neither women reported increased pain.

## 10. Summary

Nutrition modification in endometriosis has not been sufficiently studied. Further research into the effects of nutritional interventions on endometriosis is needed. Studies that have been carried out indicate the legitimacy of following a nutrition plan filled with products containing substantial amounts of antioxidants, PUFAs, vitamins D, C and E and avoiding processed foods, red meat, and animal fats. In some cases, the use of alternative diets, such as the low-FODMAP diet, a low-nickel diet, or a gluten-free diet, may be individually considered. The MD, which is among the healthiest diets, also seems to be a good solution for women with endometriosis. Table 1 shows foods that are recommended and contraindicated in endometriosis.

## 11. Limitations of This Manuscript

The limitations of this work are the absence of a structured review of scientific publications. The PRISMA guidelines were not used by the authors. A few of the tests cited were conducted only in vitro and require more detailed trials in humans.

## Figures and Tables

**Table 1:** Recommended and contraindicated foods in endometriosis [19,47,58,59,62,64].

Recommended Food Products	Contraindicated Food Products
Vegetables and fruits (source of polyphenols, phytoestrogens, resveratrol, vitamins C and E)	Processed and unprocessed red meat (effects on steroid hormones)
Spices and herbs (sources of anti-inflammatory substances)	Zoonotic fats, such as butter and lard
White and green tea (source of catechins)	Coffee (more than 300 g of caffeine per day)
Dairy products (source of calcium, vitamin D)	Highly processed products (e.g., fast food, instant and sweets)
Fish (source of omega 3 fatty acids, vitamin D)	
Vegetable oils, nuts, seeds (sources of vitamin E, omega 3 fatty acids)	

## Data Availability

Not applicable.
